# A Medical Manipulator System with Lasers in Photodynamic Therapy of Port Wine Stains

**DOI:** 10.1155/2014/384646

**Published:** 2014-08-14

**Authors:** Xingtao Wang, Chunlai Tian, Xingguang Duan, Ying Gu, Naiyan Huang

**Affiliations:** ^1^State Nuclear Power Technology Research & Development Centre, Floor 7, Building A, South Area of Future Science and Technology Park, Beiqijia Town, Changping District, State Nuclear Power Research Institute, Beijng 102209, China; ^2^Intelligent Robotics Institute, Beijing Institute of Technology, 5 Nandajie, Zhongguancun, Haidian, Beijing 100081, China; ^3^Department of Laser Medicine, Chinese People Liberation Army General Hospital, 28 Fuxing Road, Haidian, Beijing 100853, China

## Abstract

Port wine stains (PWS) are a congenital malformation and dilation of the superficial dermal capillary. Photodynamic therapy (PDT) with lasers is an effective treatment of PWS with good results. However, because the laser density is uneven and nonuniform, the treatment is carried out manually by a doctor thus providing little accuracy. Additionally, since the treatment of a single lesion can take between 30 and 60 minutes, the doctor can become fatigued after only a few applications. To assist the medical staff with this treatment method, a medical manipulator system (MMS) was built to operate the lasers. The manipulator holds the laser fiber and, using a combination of active and passive joints, the fiber can be operated automatically. In addition to the control input from the doctor over a human-computer interface, information from a binocular vision system is used to guide and supervise the operation. Clinical results are compared in nonparametric values between treatments with and without the use of the MMS. The MMS, which can significantly reduce the workload of doctors and improve the uniformity of laser irradiation, was safely and helpfully applied in PDT treatment of PWS with good therapeutic results.

## 1. Introduction

**Figure 1:**
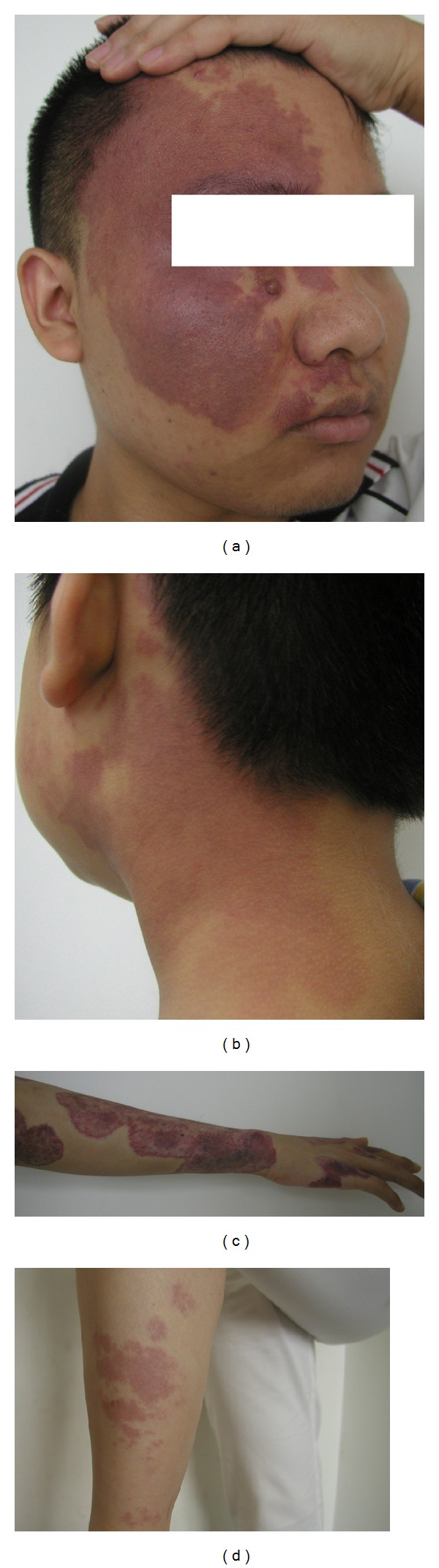
PWS symptom. (a) PWS in face. (b) PWS in neck. (c) PWS in arm and hand. (d) PWS in leg.

Port wine stains (PWS), also known as birthmarks, are a congenital expansion and malformation of the superficial dermal capillary network [1]. The morbidity rate is as high as 3–5‰ [2]. Most of PWS appear in the skin of the head, face, and neck; some appear on the legs and arms (Figure 1). PWS not only affects patients on functional aspects, but also influences patients' psychological health [3–5].

According to the histopathological changes of PWS, the main difficulty of the treatment is that the expanded and deformed capillary network in the superficial dermis should be dispelled to eliminate abnormal red lesion, while the epidermis and deep tissue should not be damaged to avoid scaring [2]. Conventional treatments, including skin grafting, irradiation, and chemical peels, are not ideal with respect to scaring and pigmentation.

Photodynamic therapy (PDT), which was started as a novel antitumor therapy in the early 1980s, is a type of laser-based treatment for PWS. In 1990, Professor Ying Gu began to study the PDT treatment of PWS and established a new PDT program for PWS. The effective rate can reach up to 98% [2, 4, 6, 7]. In the PDT treatment of PWS, a “photosensitizer” drug (such as hematoporphyrin monomethyl ether), which is highly concentrated inside PWS vessels and diffuses little to normal tissue, is injected intravenously. Then the PWS lesion is irradiated by laser. The photosensitizer is excited by the laser, producing singlet oxygen and other toxic chemical substances which will destroy the malformed vascular. The toxic chemical species are only generated in malformed vascular and have only little damage to normal skin [8].

**Figure 2:**
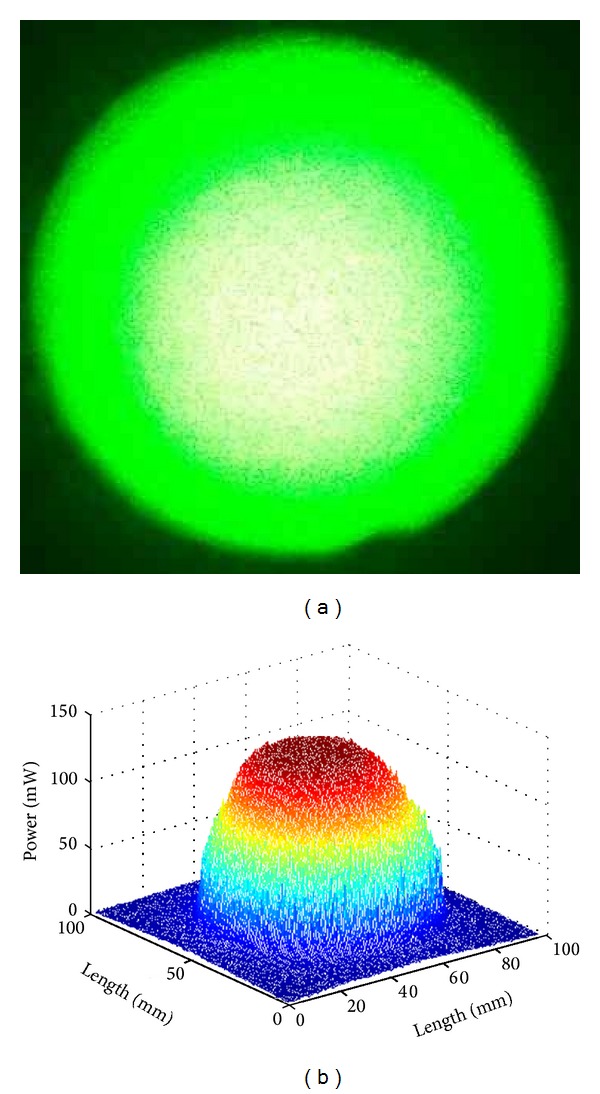
The uneven power density distribution of laser spot. (a) Laser beam image. (b) Gray handled image with MATLAB.

**Figure 3:**
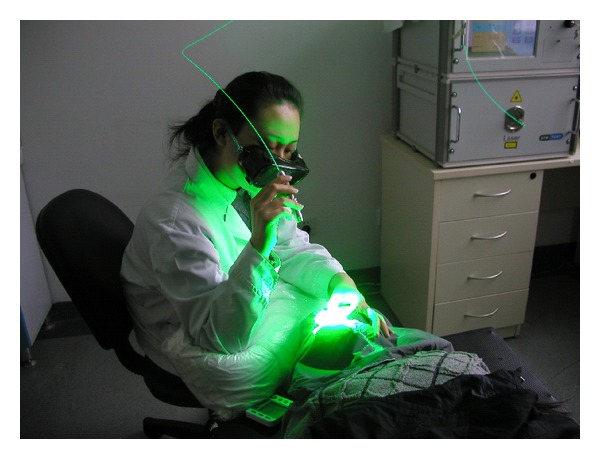
The manual operation of laser fiber by doctor.

In the PDT treatment of PWS, the correct laser power density and a uniform laser irradiation on the PWS lesion are very important to get good therapeutic effectiveness. Unfortunately, the power density of a laser spot is a Gauss distribution (Figure 2), which is uneven. For a given lesion, the laser irradiation of the central area is too high, which may cause skin burns, while the laser power density of other areas is too low, which may affect the therapeutic effectiveness. So, a uniform laser radiation, which means every section of the PWS lesion receives the same quantity of laser radiation during the PDT process which is used. A uniform radiation can be obtained by operating the laser fiber in such a way that the centre of the laser spot is kept in motion over the lesion. The operating doctor has to move the laser fiber to produce an even irradiation on the lesion and avoid skin burn (Figure 3).

The process of treating a single lesion takes about 30 to 60 minutes. This long duration manual operation is a high work load to doctors with low efficiency and accuracy. Therefore, a medical manipulator system (MMS) was developed to assist doctors in clinical treatment. Until now, 296 PWS cases have been treated using the MMS; the therapeutic results are comparable to the results of the traditional treatment. Clinical trials show that the MMS is effective and useful in PDT treatment of PWS.

## 2. Materials and Methods

### 2.1. Medical Manipulator System

**Figure 4:**
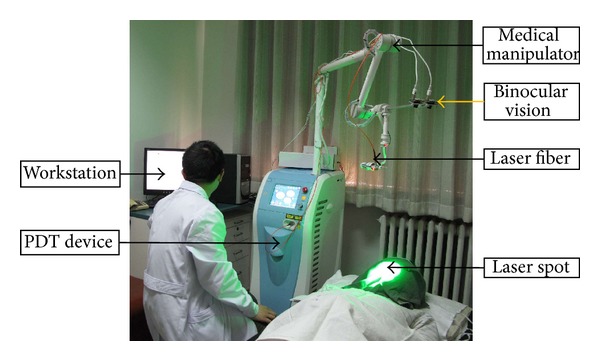
The medical manipulator system.

The MMS consists of four main components, as shown in Figure 4:

1. a PC-based workstation providing overall control using a human-computer interface, an image processing module, and an expert database subsystem;
2. a PDT device, which produces a laser beam and controls the treatment parameters, such as the laser power density and the irradiation time;
3. a medical manipulator to operate the laser fiber automatically;
4. a binocular vision system used as guidance and surveillance of the MMS.

### 2.2. Medical Manipulator

**Figure 5:**
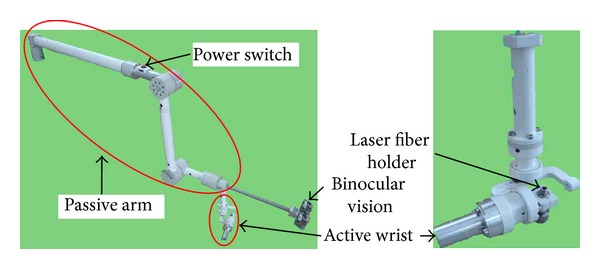
The mechanism of medical manipulator.

**Figure 6:**
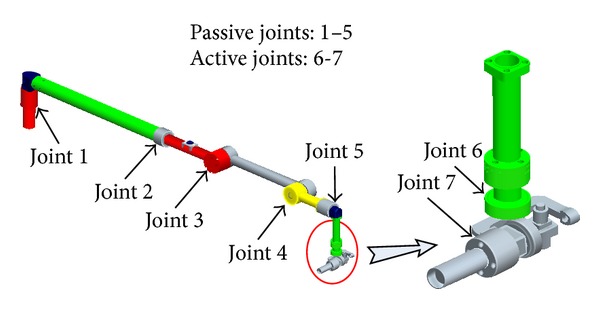
The seven-DOF arrangement of medical manipulator.

Manipulators in medical application should be safe and convenient for doctors to use [9]. Based on the analysis of treatment requirements and the application environment in Chinese PLA General Hospital (known as Beijing 301 Hospital), a novel 7-DOF medical manipulator with passive and active joints is developed (Figure 5). The medical manipulator consists of a passive arm and an active wrist. There are 5 passive joints in the arm corresponding to 5 degrees of freedom (DOF) and 2 active joints in the wrist corresponding to 2 DOF. The DOF arrangement of the medical manipulator is shown in Figure 6.

### 2.3. Passive Arm

**Figure 7:**
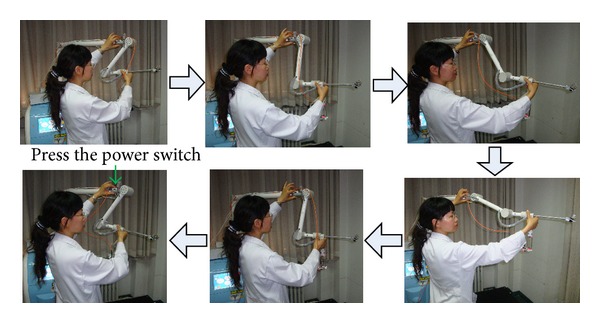
The operation of passive arm.

The 5-DOF passive arm can be adjusted manually. Once adjusted, it will hold the active wrist stationary. The arm consists of one translational DOF (Joint 2) and four rotational DOF (Figure 6). In each rotational DOF, there is an electromagnetic brake. If the power switch is pressed, all the electromagnetic brakes lose power, and the passive joints can simultaneously be freely and quickly adjusted. Once the power switch is released, the joints will be locked firmly into place again by the electromagnetic brakes (Figure 7).

The passive arm was tested by several doctors in the Department of Laser Medicine of the PLA General Hospital. According to the results, the passive arm is easy to operate, is stable to hold, and self-locks firmly; fulfilling these requirements shows that it meets all the clinical treatment requirements.

### 2.4. Active Wrist

Each of the DOF in the active wrist is controlled by DC servo motors. There are two rotational DOF, and the laser fiber is held by wrist joint 7 (Figure 6).

**Figure 8:**
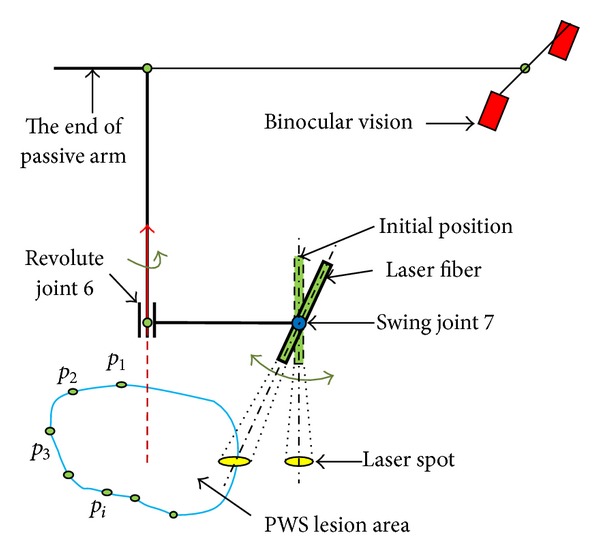
The kinematics model of medical manipulator.

In order to simplify the movement of medical manipulator, the PWS lesion is approximated to lie on a plane. By adjusting the passive arm manually, the active wrist is positioned above the PWS lesion with the guidance of binocular vision.  Figure 8 shows the kinematics model of the medical manipulator.

**Figure 9:**
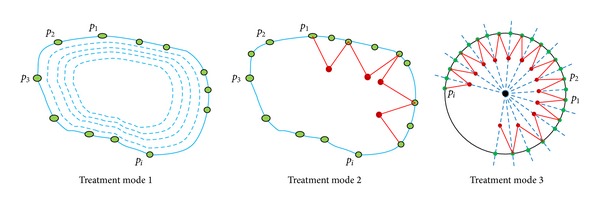
Treatment modes of medical manipulator assisted PDT for PWS.

By analysing the doctors' movement of the laser fiber during a traditional treatment, the trajectory of the laser spot's centre can be simplified and decomposed into two movements. The first is a circular movement along the circumferential direction of the lesion. The other is a swinging movement along the radial direction of the lesion. Using this analysis, three treatment modes were designed to simulate the doctors' manual operation (Figure 9).

1. Treatment mode 1: the centre of laser spot scans the lesion's perimeter. After a complete revolution, the perimeter is decreased by a specified scale and another revolution is made.
2. Treatment mode 2: while the laser scans the lesion border, the “swing joint” of the active wrist swings along the radial direction of the lesion.
3. Treatment mode 3: a circle, which includes the lesion zone, is used to approximate the lesion border. The laser moves identically to treatment mode 2.

### 2.5. Safety System

**Figure 10:**
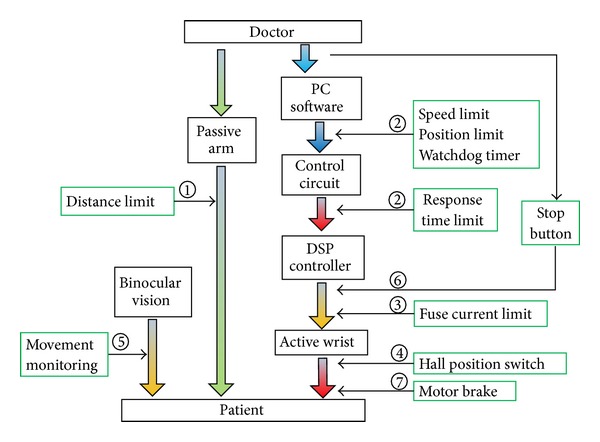
The safety precautions of MMS.

The safety system (Figure 10) was developed to avoid risks to patients under any circumstances. Besides the supervision of doctors and the use of the binocular vision system, several safety precautions were proposed including software, electrical design, and mechanical means [10–12].

1. After the passive arm is operated, if the minimum distance between the active wrist and the PWS lesion is 40 mm or less, the MMS is locked by the hardware and the software.
2. In the software program a speed limit, a position limit, and a watch dog timer are applied. Furthermore, the response time limit is set in the communication handshaking procedure between the DSP controller and the PC user software. If both of them have not received a response signal from the other within the set time (50 ms for the MMS) due to a freeze-up, runaway, or other errors, the power will be shut off.
3. Fuses are implemented to limit the current supplied to each servo motor.
4. A hall position switch is attached at each active wrist joint to limit the position.
5. The binocular vision system positions the PWS lesion and supervises the patient. If the patient moves tempestuously, an alarm is given.
6. By pressing the emergency stop button, doctor can stop the MMS at any time in case of an emergency.
7. Each servo motor is equipped with a brake so that the active joints will be locked if power is lost.

### 2.6. Clinical Treatment of PDT for PWS with MMS

#### 2.6.1. Data Collection

The MMS was applied in a clinical PDT treatment of PWS in the Chinese PLA General Hospital in 2010. A total of 296 PWS lesions were treated in patients with various ages, PWS lesion sites, and disease extents. The patients were 74 male (39.8%) and 112 were female (60.2%). The patient's ages ranged from 3 to 42 years. Some of the patients had multiple lesions (diameter > 8 cm or in more than one anatomic planes), and some of them had single lesion (diameter < 8 cm and in one anatomic plane) [5, 8]. None of the patients had received previous treatment with a complete medical record.

So far, there are no standard classification criteria for PWS. In this paper, the classification criteria which were adopted in [3, 5, 13–15] were used to classify PWS lesions into five types: Type I: pink, flat; Type II: light red, flat; Type III: dark red, flat; Type IV: purple, slightly thicker, and Type V: significantly thicker or nodular [5]. PWS lesion distribution is shown in Table 1. There are 29 (9.8%), 46 (15.6%), 117 (39.5%), 77 (26.0%), and 27 (9.1%) lesions, respectively, for each type.

#### 2.6.2. Treatment Protocols of MMS Assisted PDT Treatment of PWS

In the treatment, a PDT device (produced by Beijing Newraysing Laser Tech Co. Ltd.; power output of 5 W) was used to provide a continuous KTP laser with a wavelength of 532 nm. The laser was delivered through an optic fiber with a flat cut tip, which was held by the active wrist. The laser beam showed quasi-Gaussian output; the power density was 80–100 mW/cm^2^.

**Figure 11:**
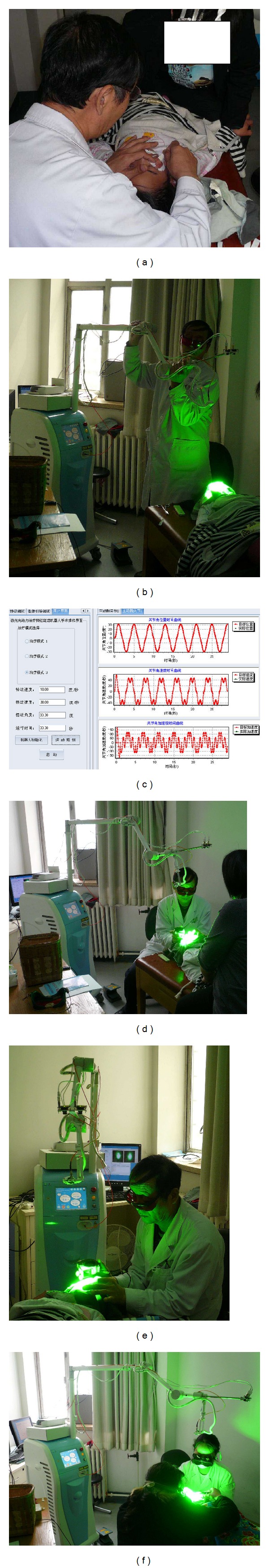
The clinical treatment with MMS. (a) Lesion selection and patient protection. (b) Passive arm operation. (c) The human-computer interface. (d) Active wrist operating the laser fiber. (e) Lesion response diagnosis. (f) Another case of MMS assisted PDT treatment of PWS.

As shown in Figure 11, after a routine allergy test, the doctor selected the lesion of the patient and protected normal skin around lesion by applying black cloth. The patient was then injected intravenously with a photosensitizer, after which the laser irradiation was performed immediately. The doctor operated the passive arm, so that the laser fiber was positioned perpendicular to the surface of the PWS lesion. The binocular vision system measured the position of the lesion and then transferred the information to workstation. The PDT device delivered the laser spot which was automatically guided by the active wrist to generate a uniform irradiation over the entire lesion. With the assistance of the MMS, the doctor had more time and energy during the treatment to diagnose the lesion response and carry out treatment accordingly. The laser irradiation lasted 20–50 minutes with a total energy density of 120–300 J/cm^2^.

The doctors can select different treatment modes in the human-computer interface. Simultaneously, the binocular vision system monitors the whole operation and the safety system of MMS guarantees patient safety during the treatment.

## 3. Results and Discussion

### 3.1. Criterions of Therapeutical Evaluation and Follow-Up

Until now, there are no standard evaluation criteria to assess the therapeutical effectiveness of the PDT treatment of PWS. In this paper, the criteria, which is introduced in [3, 5, 13–15] and is widely used by other physicians for PWS PDT evaluation, is adopted to assess the clinical outcomes of the MMS assisted PDT treatment of PWS. Therapeutic responses are recorded and defined in five grades: Grade I excellent: colour is close to normal skin colour and no scar formation; Grade II good: marked blanching, thicker lesion become flat, no scar formation; Grade III fair: partial blanching, thicker lesion becomes moderately flat; Grade IV poor: slight blanching, thicker lesion becomes slightly flat; and Grade V: no change.

The follow-up time ranged from 6 months to 3 years. The lesions were examined visually by experts of the Chinese PLA General Hospital. Photographs of pretreatment and posttreatment conditions were taken and evaluated. By comparing the lesion color after treatment to the lesion color before treatment, the grade of therapeutic responses was determined and recorded by the same doctor, who is unfamiliar with the patient group. Other responses, such as side-effects and scar formations, were also recorded.

### 3.2. Treatment Outcomes

**Figure 12:**
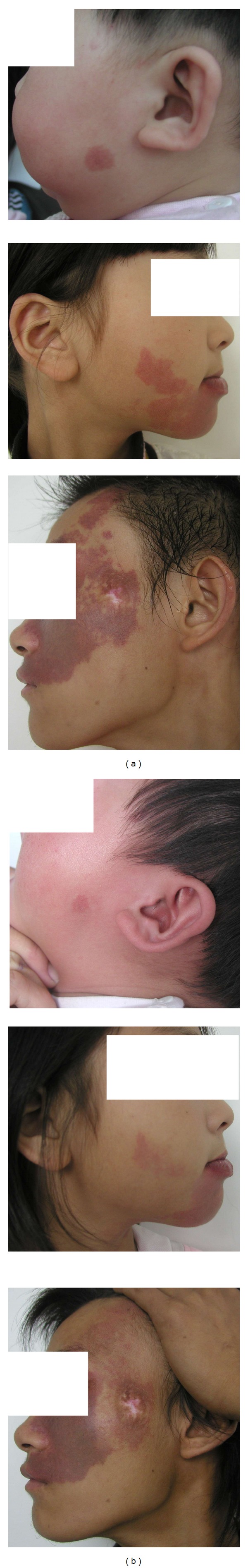
The clinical outcome of MMS assisted PDT treatment for PWS. (a) Before treatment. (b) After treatment.

Each of the patients showed different degrees of edema after treatment, but no scaring or pigmentation was observed. All of the PWS lesions showed different degrees of improvement. The clinical outcomes displayed good therapeutic results. Parts of the patients' representative photographs of before and after the PDT treatment using the MMS can be seen in Figure 12.

### 3.3. Statistical Analysis

The mechanism of PDT treatment involves the complex interactions of various factors including light, photosensitizer, oxygen, and diseased tissue. The qualitative research of a single factor cannot reveal the overall therapeutic effect of the PDT treatment of PWS [16]. The statistical comparison of nonparametric values was used to evaluate the treatment response between the MMS assisted and the traditional PDT treatment of PWS [5, 13].

As mentioned above, the therapeutic outcomes were graded as excellent, good, fair, poor, or no change. As shown in Table 2, there were 15 lesions (5.1%) which achieved excellent response, 124 lesions (41.9%) which showed good results, fair response appeared in 145 lesions (49.0%), in 11 lesions (3.7%) poor results were observed, and 1 lesion (0.3%) showed no change. In [3], the Chinese PLA General Hospital reported 1949 PWS lesions in 1385 patients treated by PDT. Excellent results were achieved in 128 lesions (6.6%), 746 lesions (38.3%) yielded good results, 923 lesions (47.4%) showed moderate results, 145 lesions (7.4%) showed poor results, and in 7 lesions (0.3%) no visible change was observed.

**Figure 13:**
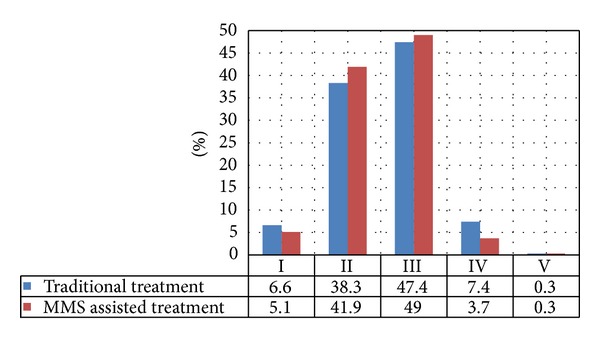
The statistical comparison of traditional and MMS assisted PDT treatment of PWS.

As shown in Figure 13, the percentage of Grade I and Grade IV outcomes in the MMS assisted treatment are lower than those in traditional treatment. Accordingly, the percentage rate of Grade II and Grade III are higher in the MMS assisted treatment, and the percentage rates of Grade V are the same.

Given the statistical analysis, although there was no significant improvement of the MMS assisted treatment method compared to the traditional treatment method, the therapeutic effectiveness of the MMS assisted treatment was almost as good as the results of traditional treatment. In particular, there were more lesions which achieved good and fair responses in the MMS assisted treatment. Considering that the traditional treatment group was achieved by experts with more than 10 years of experience in PWS-PDT treatment, the therapeutic effectiveness achieved by the MMS was also an acceptable positive result with the significant advantage of reducing doctors' work load, improving laser irradiation uniformity, and the possibility of tirelessly continuous long-time work. Therefore, the MMS is useful in assisting doctors for PDT treatment of PWS.

### 3.4. Discussion

Factors, such as depth and size of the lesion, type and dose of the photosensitizer, laser wavelength, interval time between drug injection and laser irradiation, and laser irradiation dose can make different degrees of influence on the results of PWS-PDT treatment. The following factors correlated with the clinical effect are summarized [5].

1. Depth and size of PWS lesion: a good response can be obtained when the lesion depth is less than 830 *μ*m and poor or no response is achieved at 1000 *μ*m or deeper. Larger lesions, particularly those extended to the lip, showed poor response.
2. Anatomical location of PWS lesion: generally, lesions in the forehead, lateral aspect of the cheek, neck, chest, and shoulder often showed good responses.
3. Type and dose of photosensitizer: as discussed by Gu et al. [15], different photosensitizers have different characteristics depending on the PWS lesion. The photodynamic effect can be enhanced if a higher drug dose is used, but there is a possibility of poisoning the patient.
4. Energy and fluence rate of the laser: for children and lightly colored superficial lesions, a lower fluence rate should be used in order to avoid scar formation. For thick, dark, and deep lesions, higher fluence rates should be used in order to reach the deeper lesion.
5. Uniformity of laser irradiation: in order to avoid overexposing and to prevent overheating and scaring, it is critical to insure an even light distribution.
6. Experience of doctors: during the course of treatment, the irradiation time and dose of the laser can be adjusted according to the lesion's reaction and the tissue temperature. The sensitivity to the PDT treatment is quite different for various PWS patients. The treatment experience of doctors is very important to make correct judgments, especially for the first treatment.
7. Posttreatment care: patients should avoid strong light for half to one month after the therapy. Edema and scabs should be carefully taken care of. The result of the posttreatment phase also affects the therapeutical effectiveness of the PWS-PDT treatment.

The complex influencing factors of the PWS-PDT treatment might explain why there are some poor and no change responses, and why the percentage of Grade I results is lower than in the traditional treatment. Although the MMS can only optimize some of the listed factors, considering the advantages (discussed following) of the MMS and the therapeutical evaluation (discussed above), the MMS is feasible and useful in assisting doctors giving PDT treatment for PWS.

The advantages and limitations of MMS are summarized below.

*Advantages*

1. Improving the uniformity of laser irradiation: the MMS has the same advantages as a medical robot: high positioning accuracy, firm grip, and smooth movement. In the MMS, a binocular vision system can position the PWS lesion precisely; the medical manipulator can firmly hold the laser fiber and operate it smoothly and with high accuracy. The MMS improves the uniformity of laser irradiation which ensures that every section of the PWS lesion receives the same quantity of laser radiation during the PDT process. Additionally, the system avoids overexposing or overheating the lesion.
2. Lightening doctors' workload: the laser fiber is automatically operated by an active wrist in the MMS, which lightens the doctors' workload. This makes it possible for the doctor to devote more energy to diagnose the lesion's response and pay more attention on the patient's comfort during the PDT treatment, which is clinically favorable.
3. Treating more patients: the MMS can operate the laser fiber continuously without rest, so that doctors have the opportunity to take care of a larger amount of patients.
4. Reducing influence of personal factors: in a conventional manually operated treatment, laser irradiation of a PWS lesion is arbitrary and nonuniform. In the MMS assisted treatment, the movement of the laser fiber can be quantified, which reduces the impact of human factors and is good for therapeutical evaluation.

*Limitations*

1. A major problem is that if the patient suddenly moves too much, the binocular vision system will lose the position of the PWS lesion. The positioning algorithm is not stable enough under the condition of high-intensity laser irradiation.
2. The workspace of the active wrist is limited. Sometimes, during a clinical treatment, the laser fiber interfered with the active wrist during the swinging movement, especially when the PWS lesion had a large surface area.
3. The active wrist does not have enough DOF. It is not possible for a patient to lie on a bed motionless throughout the treatment. Due to pain or an uncomfortable posture, the PWS lesion was turned a certain angle by the movement of patient. Two DOF are not enough to follow the lesion movement automatically.
4. During the clinic treatment, doctors needed more time to prepare the MMS. The human-computer interface was not convenient enough for doctors to use. The process of operating the MMS is still complex; the initial position of laser spot needed to be manually adjusted by doctors using the computer interface.

## 4. Conclusions

In this paper, a medical manipulator system is put forward to assist doctors for the PDT treatment of PWS. The uniformity of laser irradiation can be improved by precise and stable movement of a medical manipulator. The doctors' workload can be lightened by operating the laser fiber with the MMS instead of moving it manually. More patients can be treated by the continuous and tireless work of the MMS. Clinical results show that the MMS is useful in PDT treatment of PWS; it can improve the therapeutical effectiveness and doctor satisfaction. The MMS appears to have a promising future, and it hopefully will become an objective and reliable PWS therapy machine in the future.

## Figures and Tables

**Table 1:** Clinical characteristic.

Number of patients	Number of Lesions	Gender		Age	Lesion type				
		Male	Female		I	II	III	IV	V
186	296	74 (39.8%)	112 (60.2%)	3–42	29 (9.8%)	46 (15.6%)	117 (39.5%)	77 (26.0%)	27 (9.1%)

**Table 2:** The statistical results of traditional and MMS assisted PDT treatment of PWS.

Grades	Traditional PDT treatment of PWS		MMS assisted PDT treatment of PWS	
	Number of lesions	Percentage (%)	Number of lesions	Percentage (%)
I	128	6.6	15	5.1
II	746	38.3	124	41.9
III	923	47.4	145	49.0
IV	145	7.4	11	3.7
V	7	0.3	1	0.3

Total of lesions	1949		296	
